# Healthcare-associated infections

**DOI:** 10.3205/dgkh000605

**Published:** 2025-12-05

**Authors:** Gargee Anand, Rijhul Lahariya

**Affiliations:** 1 All India Institute of Medical Sciences, Patna, Bihar, India

**Keywords:** healthcare-associated infections, antimicrobial resistance, infection prevention and control, antimicrobial stewardship, healthcare-associated pathogens, multidrug-resistant organisms

## Abstract

**Background and purpose:**

Healthcare-associated infections (HAIs) continue to be a leading cause of morbidity, mortality, and increased healthcare costs worldwide. Despite advances in infection prevention, the burden of HAIs remains high, exacerbated by the rise of antimicrobial resistance (AMR) and the growing complexity of patient care. Effective prevention strategies are critical to reducing HAI rates and improving patient outcomes. This review aims to highlight the clinical significance of HAIs, explore their impact on patient safety, and underscore the necessity for robust surveillance and infection control (IC) programs across healthcare settings.

**Results:**

The study found that HAIs continue to affect a significant proportion of hospitalized patients, with invasive devices and antimicrobial-resistant pathogens being key contributors. Surveillance systems, when combined with targeted IC protocols and continuous staff education, can reduce HAI incidence and improve patient safety. Moreover, the implementation of antimicrobial stewardship programs and proper hygiene practices along with emerging technologies plays a pivotal role in curbing the spread of resistant organisms.

**Conclusion:**

Healthcare systems must prioritize HAI prevention to safeguard patient safety, reduce treatment costs, and combat the growing threat of AMR.

## Zusammenfassung

**Hintergrund und Zielsetzung:**

Healthcare-assoziierte Infektionen (HAI) sind nach wie vor eine der Hauptursachen für Morbidität, Mortalität und erhöhte Gesundheitskosten weltweit. Trotz Fortschritten in der Infektionsprävention bleibt die Belastung durch HAI hoch, was durch die Zunahme antimikrobieller Resistenzen (AMR) und die zunehmende Komplexität der Patientenversorgung noch verschärft wird. Effektive Präventionsstrategien sind entscheidend für die Reduzierung der HAI-Raten und die Verbesserung der Heilungsergebnisse. Ziel des Reviews ist es, die klinische Bedeutung von HAI hervorzuheben, ihre Auswirkungen auf die Patientensicherheit zu analysieren und die Notwendigkeit robuster Surveillance- und IC-Programme im Gesundheitswesen zu unterstreichen.

**Ergebnisse:**

Die Studie ergab, dass weiterhin ein erheblicher Anteil der Krankenhauspatienten von HAI betroffen ist, wobei invasive Devices und multiresistente Erreger (MRE) die Hauptursachen sind. Die Surveillance kann in Kombination mit Protokollen zur gezielten Infektionskontrolle (IC) und mit kontinuierlicher Schulung des Personals die HAI-Inzidenz reduzieren und die Patientensicherheit verbessern. Darüber hinaus spielt der Einsatz antimikrobieller Stewardship-Programme und angemessener Hygienepraktiken zusammen mit neuen Technologien eine entscheidende Rolle bei der Eindämmung von MRE.

**Schlussfolgerung:**

Gesundheitssysteme müssen der HAI-Prävention Priorität einräumen, um die Patientensicherheit zu gewährleisten, Behandlungskosten zu senken und die wachsende Bedrohung durch AMR zu bekämpfen.

## Introduction

The Centers for Disease Control and Prevention (CDC) define a healthcare-associated infection (HAI) as one that occurs on or after the 3^rd^ day of a patient’s hospital stay, with the admission day counting as day 1 [1]. The CDC play a crucial role in addressing HAIs by providing national leadership in surveillance, outbreak investigations, laboratory research, and prevention efforts, with their National Healthcare Safety Network (NHSN) serving as the USA’s most widely used HAI tracking system and work toward eliminating these infections [2], [3]. The World Health Organization (WHO) defines a HAI, also known as a nosocomial infection, as an infection occurring in a patient during their stay at a healthcare facility, starting on or after the 3^rd^ day of admission, and can develop while receiving care or even after discharge [4]. This makes HAIs a significant global concern, with the WHO highlighting their widespread occurrence in healthcare facilities worldwide, where they pose a constant health threat by prolonging hospital stays, escalating antimicrobial resistance (AMR), and placing significant financial burdens on health systems, patients, and their families, while causing preventable deaths [4], [5]. The WHO emphasizes the urgent need to prevent and control HAIs, as they are major contributors to AMR, which disproportionately impacts low-resource settings, with up to 3.5 million deaths annually without immediate intervention [5]. In the USA, HAIs account for approximately 2 million cases and 99,000 deaths annually, making them the fifth leading cause of death in acute-care hospitals, while in developing countries, HAI prevalence can soar to 30%–50% [6]. The treatment of HAIs often requires broad-spectrum antibiotics, which increases the risk of multidrug-resistant organisms (MDRO), causing over a million deaths annually worldwide [7]. In addition to the health impact, HAIs also impose a heavy financial burden on healthcare systems as well as on patients, diverting essential resources from other critical areas of patient care [8]. Infected patients incur 2.8-fold higher costs and their hospital stays are 2.5-fold longer, adding an average of 11 extra days to their stay [9]. The most critical HAIs are those linked to invasive devices, including central line-associated bloodstream infections (CLABSI), catheter-associated urinary tract infections (CAUTI), ventilator-associated pneumonia (VAP), and surgical site infections (SSI) [10]. Preventing and controlling HAIs is vital for improving patient outcomes and combating AMR. Strengthening infection control (IC) measures must be a priority in healthcare settings before resorting to antibiotics.

## Global HAI prevalence and key pathogens responsible

According to the WHO, HAIs are significantly more prevalent in low- and middle-income countries (LMICs) than in high-income countries [4]. While HAIs are the most frequent adverse event in healthcare, the true global burden remains unclear due to difficulties in obtaining reliable data [4]. The WHO divides the globe into six regions to streamline work and improve healthcare coordination. These regions are: African Region (AFR), Region of the Americas (AMR), South-East Asia Region (SEAR), European Region (EUR), Eastern Mediterranean Region (EMR), and Western Pacific Region (WPR) [11]. HAIs caused by various pathogens, and their prevalence in specific infection types is summarized in Table 1 (Tab. 1) [12]. 

## Surveillance of HAI

HAI surveillance is very crucial for assessing their burden, identifying high-risk populations, and guiding infection prevention efforts. It relies on standardized case definitions, ensuring accurate data to effectively reduce HAI incidence and improve patient safety globally [13]. The most widely used HAI surveillance criteria are those established by the CDC NHSN, the ECDC and the WHO [14], [15], [16]. While all three organizations aim to monitor and reduce the burden of HAIs, use standardized criteria for HAI surveillance, and focus on identifying and reporting infections acquired in healthcare settings, they differ in scope and focus [17]. The CDC NHSN focuses on specific infections in the U.S. with detailed reporting, the ECDC harmonizes surveillance across European countries, and the WHO provides a global framework for infection prevention and HAI surveillance with a broader approach to strengthening health systems [17].

## Factors contributing to HAI

### Invasive devices

Invasive devices such as endotracheal tubes, urinary catheters, and central venous catheters are shown to be linked to higher rates of HAIs in ICU settings [18], [19]. Invasive devices bypass the body’s natural defense and create entry points for infections, creating an easier pathway for microorganisms to enter and transfer between different parts of the patient’s body, from healthcare workers (HCWs) to patients, or even between patients [20], [21].

### Biofilms

Once medical devices such as catheters or ventilators are inserted into the body, a protein-rich coating forms around them, providing a surface for microorganisms like *Staphylococcus* *aureus*, *Escherichia coli*, *Pseudomonas aeruginosa* and *Candida* spp. to attach and thrive [22], [23]. Over time, these bacteria multiply and create biofilms, which mature, break off and spread, entering the bloodstream and potentially leading to severe infections there and elsewhere, e.g.in the urinary tract [22]. These biofilms make pathogens highly resistant to antibiotics and the body’s own immune system, leading to persistent and hard-to-treat infections [24], biofilms are thus also a major cause of infection recurrence [25]. In many cases, the only way to treat these infections is by removing the infected device [26]. 

### Environmental factors

Pathogens spread easily in the hospital environment through contaminated hands of HCWs, equipment, and surfaces, especially in patient rooms. Microorganisms such as *Staphylococcus* *aureus*, vancomycin-resistant *Enterococcus* (VRE), *Clostridium difficile*, *Pseudomonas* *aeruginosa*, and *Acinetobacter* spp. survive for hours to months, depending on factors like location, biofilm formation, and resistance to cleaning products. Moreover, conditions of the hospital environment, for instance, contaminated air-conditioning systems and the layout of healthcare facilities (e.g., crowded units with beds placed close together, installing new wiring for information systems, removing old broken sinks, and fixing elevator shafts), also play a significant role in the transmission of HAIs [10], [27], [28], [29], [30], [31]. These factors, along with staffing issues (inadequate nurse-to-patient ratios and the absence of effective IC programs to name just two), can promote the spread of infections within healthcare settings [10].

Patient-related factors: The severity of pre-existing or underlying illness, use of immunosuppressive medications, and longer duration of hospital stays, contribute to an increased risk of HAIs by weakening the body’s ability to fight off infections, making patients more vulnerable to pathogens [10].

### Antimicrobial resistance in context with HAI

AMR occurs when pathogens no longer respond to medicines, making infections harder to treat and increasing the risk of severe illness and death. The CDC estimates that 1 in 31 patients will contract a HAI while being treated for an unrelated condition [32]. HAIs are driving the spread of AMR, which the WHO warns could lead to 10 million deaths a year by 2050, surpassing cancer, while the World Bank warns it could add $1 trillion to global healthcare costs [32]. While AMR is a natural process, misuse and overuse of antibiotics in humans, livestock, and plants have contributed to the emergence and spread of AMR microorganisms, making HAIs difficult to treat. AMR is a global threat to treating infections and performing life-saving procedures [33]. Antibiotic-resistant bacteria, including the high-risk ‘ESKAPE’ pathogens, have significantly worsened the global burden of HAIs, especially in developing countries, due to their multidrug resistance and high virulence, making treatment increasingly difficult [34], [35], [36], [37]. The burden of HAIs is closely linked to AMR, with treatment often requiring broad-spectrum antibiotics due to increased risk of MDR organisms [7]. 

## Biofilm and AMR

Biofilms contribute to AMR through several mechanisms:

- Physical barrier: The slimy matrix of biofilms prevents antibiotics from effectively reaching bacterial cells, reducing drugs' ability to kill or inhibit bacteria inside the biofilm [26].
- Recalcitrance: Biofilm-forming bacteria exhibit recalcitrance, meaning they can survive even in the presence of high doses of antibiotics. This is due to the protective environment created by the biofilm structure, which helps bacteria thrive despite drug exposure [26].
- Gene transfer: Biofilms facilitate exchange of AMR genes between bacteria, enhancing the spread of resistance within and between bacterial species [26].
- Adaptive resistance: Within the biofilm, bacteria can employ various resistance mechanisms like target modification, efflux pumps, and antibiotic inactivation, allowing them to evade both antimicrobial treatments and host immune responses [26].

## Spread of MDRO

- **Person-to-person transmission:** Pathogens can spread from one person to another through surfaces (e.g., bedrails) or the hands of HCWs if proper IC measures are not followed [38].
- **Medical procedures and devices:** Invasive procedures like surgery and the use of medical devices (e.g., catheters, ventilators) can allow resistant pathogens to enter the body and cause infections [38].
- **Patient transfers:** Resistant pathogens can spread when patients are transferred between healthcare facilities or sent home [38].
- **Fecal waste:** Fecal matter can carry traces of antimicrobials and AMR pathogens. These microorganisms can survive in plumbing and spread through sinks, toilets, and wastewater systems [38].
- **Challenges in wastewater management:** Resistant microorganisms in healthcare wastewater, especially from inpatient facilities, are hard to manage and can spread to treatment plants [38].
- **Community spread: **When resistant pathogens from healthcare settings spill over into communities, they become much harder to control [38].

## Emerging pathogens causing HAI

A growing concern today is the increase in HAIs driven by the spread of emerging drug-resistant pathogens [39]. Emerging infectious diseases are those that have recently appeared in a population or are rapidly spreading in frequency or location [32]. While many microbes contribute to AMR, the CDC currently lists *Clostridioides difficile*, carbapenem-resistant Enterobacteriaceae, drug-resistant *Neisseria gonorrhoeae*, *Candida auris*, and carbapenem-resistant *Acinetobacter* spp. as the most urgent threats [32], [40]. *Chryseobacterium indologenes*, a hospital contaminant, is emerging as causative agent of HAIs because of increased use of carbapenems and colistin, as it is intrinsically resistance to these antibiotics [41]. Rare but alarming pathogens such as *Stenotrophomonas maltophilia*, Shewanella* putrefaciens*, *Ralstonia pickettii*, *Providencia*, *Morganella*, *Nocardia*, *Elizabethkingia*, *Proteus*, and *Burkholderia* spp. are also emerging as major causes of HAIs, linked to high mortality rates and public health risks [42], [43]. *Stenotrophomonas maltophilia* along with *Burkholderia cepacia*, *Ralstonia pickettii*, and *Elizabethkingia* *meningoseptica* present serious treatment challenges due to their resistance to polymyxins and colistin, while *Elizabethkingia* spp. are especially tough to treat, being resistant to Gram-negative treatments but susceptible to Gram-positive ones (e.g., vancomycin) [42], [44], [45].

## HAI prevention and control

HAIs remain a significant challenge, making infection prevention and control (IPC) essential for protecting patients and HCWs. IPC ensures safety and quality, especially in LMICs facing additional challenges. Strengthening IPC is key to achieving high-quality, safe healthcare and preventing patient harm [46]. The key strategies for HAI prevention and control are presented in the following.

### Hand hygiene (HH)

Noncompliance with HH among HCWs is the single most crucial and global issue contributing to HAIs, requiring standardized, multimodal policies, continuous education, and regular monitoring to improve adherence and patient safety [47], [48]. Proper HH with alcohol-based hand rubs before and after handling catheter sites, including insertion, maintenance, or dressing, is the first line of defense in preventing and reducing HAIs, ensuring patient safety and promoting a healthier healthcare environment [49], [50]. Each year, the WHO’s “SAVE LIVES: Clean Your Hands” campaign urges HCWs and patients to prioritize HH, a practice that has become even more vital after the COVID-19 pandemic [46].

### Personal protective equipment

Proper use of maximal sterile barrier precautions, such as sterile gloves, sterile gowns, cap and masks, is crucial for preventing the spread of HAIs, especially when handling potentially infectious materials [21].

### Aseptic techniques

Proper aseptic technique is vital in preventing HAIs during catheter insertion. Non-sterile gloves with a “no-touch” technique suffice for peripheral venous catheters, but sterile gloves are essential for central catheters [49]. HCWs must be trained in aseptic protocols, as breaches increase infection risks and costs. Routine audits by infectious disease teams are crucial to ensure compliance and enhance patient safety. Prioritizing aseptic technique helps prevent HAIs and improves patient outcomes [51].

### Antimicrobial stewardship (AMS)

With rising antibiotic resistance and limited new drugs, AMS emphasizes the prudent use of antibiotics to improve outcomes, reduce resistance, and cut costs. AMS ensures the right antibiotic, dose, and duration to minimize toxicity and resistance [47]. The CDC’s Core Elements highlight key strategies: leadership commitment, accountability, drug expertise, action, tracking, reporting, and education. The three primary goals of AMS are:

1. ensuring rational and targeted antimicrobial use;
2. halting overuse and misuse; and
3. slowing resistance spread. Two main approaches include restricting prescriptive authority and using culture tests to guide treatment, ensuring appropriate use [52], [53]. AMS programs enhance infection cure rates, reduce harm, and help combat AMR [54].

### Disinfecting surface cleaning

Inadequate cleaning of hospital surfaces spreads harmful pathogens such as methicillin-resistant *Staphylococcus aureus* (MRSA), VRE, *Clostridium difficile*, *Acinetobacter* spp., and norovirus. Thorough cleaning is essential to reduce infection and maintain safety standards [54], [55], [56].

### Surveillance and monitoring

HAI surveillance is crucial for guiding effective IPC strategies, and should follow national guidelines. However, these can be customized to each facility’s needs and resources. Both active surveillance (e.g., screening for MRSA, carbapenem-resistant Enterobacteriaceae) and passive surveillance (e.g., lab results, patient records) are essential, tailored to each facility’s needs. Early detection of outbreaks and resistance trends enhances patient safety, reduces infections, and strengthens IC efforts [57].

### Education and training

IPC education is essential for all HCWs, from the newly hired to experienced staff. Ongoing training and audits ensure that staff are up-to-date on best practices and SOPs for IC, including HH, aseptic techniques for invasive procedures, as well as key bundles for CLABSI, CAUTI and VAP, environmental hygiene, reprocessing of medical devices, laundry hygiene, waste management, and water and air hygiene [54]. Training should be hands-on, using various methods to engage staff and improve knowledge [58]. Regular updates to training programs tailored to local needs ensure compliance and reduce HAIs, safeguarding patients and HCWs while enhancing care quality [57]. 

## Innovations in HAI prevention and control

Recent innovations in IPC are making a transformative impact in the fight against HAIs. Below are examples of cutting-edge technologies and approaches that have shown promise in HAI prevention:

- **Automated hand hygiene surveillance systems (AHHSS): **HH compliance is crucial in preventing HAIs. AHHSS address the challenges of traditional monitoring methods, such as observer bias, the Hawthorne effect and inconsistent training. A recent study showed effectiveness of AHHSS in improving HH compliance, with post-intervention compliance improving from 66%–95% to 77%–90% [59], [60]. AHHSS provide real-time monitoring, instant feedback, and consistent tracking across units, reducing human error and bias. They also integrate with cloud technology, offering easier data access for faster decision-making with minimal IT support [61].
- **Advanced disinfection technologies: **UV-A light offers safe, continuous decontamination in healthcare settings, significantly reducing pathogens such as *E. coli*, MRSA, and *Candida auris* without harming patients or staff. Its use in occupied areas can enhance IC [62]. UV-C light effectively targets MDR pathogens – for instance, MRSA, VRE, and *C. difficile* – by inducing DNA damage that prevents microbial replication, achieving up to a 4-log reduction in bacterial colonies. The drawback is that it requires vacant rooms, unlike UV-A [63], [64], [65]. Novel technologies allow the presence of persons in rooms while UV disinfection is taking place [66]. Further research is needed to optimize the effectiveness against a broader range of pathogens to fully validate its role in clinical environments.
- **Automated reprocessing systems:** Improperly cleaned reusable medical devices are a major source of HAIs. Cleaning – the first step, often overlooked – is critical to prevent biofilm formation and ensure effective disinfection. Manual cleaning is error-prone and reliant on staff training, leading to missed steps, especially in understaffed hospitals. Automated reprocessors improve cleaning accuracy and consistency, reducing human error, easing staff strain, and enhancing patient safety. Studies show reprocessing accuracy soars from 1.4% with manual methods to 75.4% with automation [67], [68].
- **Artificial intelligence (AI) and machine learning: **HAI prevention through innovations that detect infections before clinical signs appear can stratify patient risk, automate surveillance, include smart monitoring systems, intelligent disinfection robots and enhanced disinfection protocols, all of which shifts IC from reactive to preventive, thus reducing mortality and healthcare costs while improving clinical efficiency [69].
- **Virtual reality (VR) and augmented reality (AR): **VR and AR transform IC training by offering immersive, engaging, and realistic experiences that enhance learning, retention, and engagement. These scalable, cost-effective tools boost HCW performance and help reduce HAIs [70].

## Conclusion

HAIs are a critical clinical issue that directly impacts patient outcomes, healthcare resources, and overall healthcare quality. These infections not only prolong hospital stays but also increase the risk of complications, contribute to rising healthcare costs, and in some cases, lead to preventable deaths. Clinically, HAIs are major cause of morbidity and mortality, especially among immunocompromised patients or those undergoing invasive procedures.

The rise of AMR complicates their management, making timely diagnosis, effective treatment, and IC efforts more challenging. This makes the prevention of HAIs a clinical priority for HCWs at all levels. Understanding the scope of HAIs and implementing evidence-based IPC strategies can significantly improve patient safety, reduce treatment failures, and decrease unnecessary hospital readmissions. Emerging technologies, such as AHHSS, advanced disinfection methods, and AI have shown promise in enhancing IC measures and improving patient outcomes.

This study is crucial for all HCWs, as it emphasizes the importance of structured surveillance, active and passive monitoring, and continuous education on IC practices. By disseminating this information, we can ensure that clinicians, infection prevention teams, and hospital administrators are better equipped to tackle HAIs, improve patient outcomes, and minimize the growing threat of AMR. Knowledge of these issues should be integral to clinical practice, as it empowers healthcare teams to take actionable steps to reduce infections, enhance patient care, and ultimately save lives.

## Notes

### Authors’ ORCIDs

- Anand G: https://orcid.org/0009-0008-0473-389X
- Lahariya R: https://orcid.org/0009-0003-5769-4509

### Funding

None. 

### Competing interests

The authors declare that they have no competing interests.

## Figures and Tables

**Table 1:**
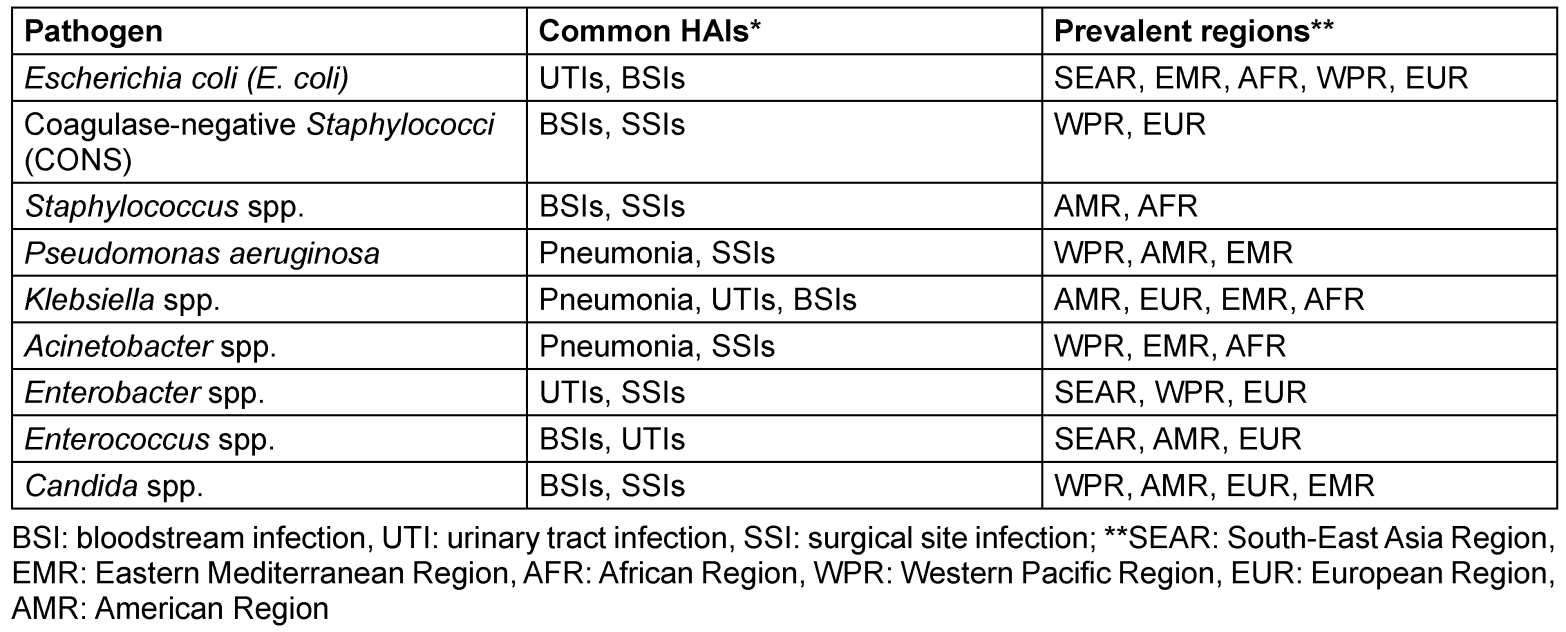
Prevalence of pathogens in healthcare-associated infections across different regions
